# VO_2_ and VCO_2_ variabilities through indirect calorimetry instrumentation

**DOI:** 10.1186/2193-1801-2-688

**Published:** 2013-12-23

**Authors:** Miguel Cadena-Méndez, Boris Escalante-Ramírez, Joaquín Azpiroz-Leehan, Oscar Infante-Vázquez

**Affiliations:** Centro de Investigación en Instrumentación e Imagenología Médica, Departamento de Ing Eléctrica, Universidad Autónoma Metropolitana-Iztapalapa, Mexico City, DF México; Departamento de Procesamiento de Señales, Facultad de Ingeniería, Universidad Nacional Autónoma de México, Ciudad Universitaria, Tlalpan, Mexico City, DF México; Departamento de Instrumentación Electromecánica, Instituto Nacional de Cardiología Ignacio Chávez, Mexico City, DF México; Research Center in Instrumentation and Medical Imaging, Departamento de Ingeniería Eléctrica, Universidad Autónoma Metropolitana-Iztapalapa, San Rafael Atlixco 186 Iztapalapa, Distrito Federal, CP 09340 Mexico City, México

**Keywords:** VO2 and VCO2 variabilities, Gas exchange variability, Variability, Open circuit hybrid calorimeter, VO2 and VCO2 power spectrum

## Abstract

The aim of this paper is to understand how to measure the VO_2_ and VCO_2_ variabilities in indirect calorimetry (IC) since we believe they can explain the high variation in the resting energy expenditure (REE) estimation. We propose that variabilities should be separately measured from the VO_2_ and VCO_2_ averages to understand technological differences among metabolic monitors when they estimate the REE. To prove this hypothesis the mixing chamber (MC) and the breath-by-breath (BbB) techniques measured the VO_2_ and VCO_2_ averages and their variabilities. Variances and power spectrum energies in the 0–0.5 Hertz band were measured to establish technique differences in steady and non-steady state. A hybrid calorimeter with both IC techniques studied a population of 15 volunteers that underwent the clino-orthostatic maneuver in order to produce the two physiological stages. The results showed that inter-individual VO_2_ and VCO_2_ variabilities measured as variances were negligible using the MC while variabilities measured as spectral energies using the BbB underwent 71 and 56% (p < 0.05), increase respectively. Additionally, the energy analysis showed an unexpected cyclic rhythm at 0.025 Hertz only during the orthostatic stage, which is new physiological information, not reported previusly. The VO_2_ and VCO_2_ inter-individual averages increased to 63 and 39% by the MC (p < 0.05) and 32 and 40% using the BbB (p < 0.1), respectively, without noticeable statistical differences among techniques. The conclusions are: (a) metabolic monitors should simultaneously include the MC and the BbB techniques to correctly interpret the steady or non-steady state variabilities effect in the REE estimation, (b) the MC is the appropriate technique to compute averages since it behaves as a low-pass filter that minimizes variances, (c) the BbB is the ideal technique to measure the variabilities since it can work as a high-pass filter to generate discrete time series able to accomplish spectral analysis, and (d) the new physiological information in the VO_2_ and VCO_2_ variabilities can help to understand why metabolic monitors with dissimilar IC techniques give different results in the REE estimation.

## Background

Indirect calorimetry (IC) has been considered by physicians, clinical nutritionists, and researchers as the gold standard to estimate the resting energy expenditure (REE) and the metabolic substrate utilization in humans (Ferrannini 1988; Branson and Joahanningam 2004). The IC technique has been implemented through the respiratory gas exchange measurement in order to calculate mainly the averages of the oxygen consumption (VO_2_) and carbon dioxide production (VCO_2_) using different kinds of metabolic monitors with dissimilar instrumental techniques that can possibly produce different results in the estimation of the REE (McClave and Snider 1992).

The general clinical view assumes that the REE estimation inconsistency is the result of uncertainties in the instruments at the time they compute de VO_2_ and VCO_2_ averages in ambulatory or critical care patients (Matarese 1997). However, this high REE variation is not only consequence of instrumental precision problem since it has different causes other than sensors and electronics noise.

We believe that the variation in the REE estimation mainly depends on how the VO_2_ and VCO_2_ variabilities are detected and measured during the standard VO_2_ and VCO_2_ average estimations. The variabilities basically depend on the patients’ physiological state, since each one responds differently, during the IC studies, to the stimuli produced by their diseases, environmental stress conditions (temperature, noise, humidity, etc.) and to the metabolic monitoring connector devices such as face masks, canopies and mouth connectors (Garby and Lammert 1994).

The problem is that the commercial metabolic monitors do not separate and measure the VO_2_ and VCO_2_ variabilities from their average computation in order to correctly interpret whether these variabilities are noise, artifacts, or if they can contain physiological information that may help to understand the variation in the REE estimation (Karsegard et al. 2010; Sundstrom et al. 2013). How to measure the VO_2_ and VCO_2_ variabilities in steady or non-steady state is an open question since metabolic monitors have been designed only to compute the VO_2_ and VCO_2_ averages in IC clinical studies which respond to a different type of patient needs such as the pediatric, ambulatory, intensive care or those subjects in free movement conditions (Simonson and DeFronzo 1990).

The premise is that the metabolic monitors use different instrumental IC techniques which can filter or enhance the VO_2_ and VCO_2_ variabilities depending on the design of the pneumatic hardware. For instance, in calorimeters with mass exchange, which can be considered as an open-design pneumatic system, the VO_2_ and VCO_2_ measurements of averages are carried out faster even in a portable manner but with more sensitivity to detect the embedded variabilities (Brown et al. 1984; Myers et al. 1990). On the other hand, in metabolic monitors with closed-design pneumatic circuit the VO_2_ and VCO_2_ variabilities are filtered because their frequency bandwidth response is probably limited by the great size of their instrumental set-ups (Holdy 2004). In both cases, no study exists that would suggest which type of pneumatic hardware would be more suitable to measure the variabilities separately. Thus, metabolic monitor manufacturers are free to promote their own hardware designs, which have the sole purpose to obtain the best estimation of the VO_2_ and VCO_2_ averages.

Currently most of the commercial metabolic monitors in use are open circuit systems with the capability to perform IC studies in the range of 15–20 minutes. The bench performance in their accuracy has been evaluated in the range of 2 to 3%, which does not explain the high variation in the REE estimation, in critical care and ambulatory patients (Reeves et al. 2004 and Cooper et al. 2009). The REE variation has been reported as inter-subject or intra-subject variability despite following the clinical guide for standardizing IC studies. Therefore, we assume this guide is only focused on controlling the patient’s steady state in order to facilitate the prediction of the REE in 24 hours without taking into account unsteady patients (AARC Clinical Practice Guideline 2004). The second assumption is that the IC studies in non-steady state, as part of the clinical routine, are accountable for the high variability in the REE estimation (Wessel et al. 1979). Then, current guidance has a limited scope since it only describes measurement conditions when patients are in steady state such that their physiological reaction to thermal and noise environment is monitored and controlled only through the measurement of the variation coefficient (VC) that should be maintained by no more than ± 5% in any single 5 minute measurement interval (McClave et al. 2003).

Consequently, any proposal for a new IC clinical guide with the capability to define studies in steady and non-steady state should consider the following facts: (a) only 54% of ambulatory patients who are submitted to REE estimation comply with the current VC defined threshold (McClave and Snider 1992; Damask et al. 1983). (b) Different IC studies in ambulatory subjects have reported intraindividual REE variability of 12.5% and 23% during two consecutive days and over a period of 2 years, respectively (Mindy et al. 1986). (c) Studies in critically ill patients have reported inter-individual REE variability up to 64% with different metabolic monitors (Schadewaldt et al. 2013). (d) Investigations have shown increments beyond 15% in the VC due to what is called *physiological reactivity* which is associated to the effect of the connecting devices such as face masks, mouth connectors and canopies (McAnena et al. 1986; Segal 1987; Isbell et al. 1991; Forse 1993). (e) Metabolic monitors for patients in a hospital or the ambulatory environment having different pneumatic techniques and algorithms have not been evaluated to perform IC studies in steady and non-steady state conditions, and (f) the most frequently used open circuit calorimeters have two different design concepts known as the breath by breath (BbB), and the mixing chamber (MC) techniques that may produce different REE estimation results (Faver et al. 1998; Arch et al. 2006).

Observations, such as those previously mentioned, have triggered our main assumption that the VO_2_ and VCO_2_ variability analysis can help to understand the variation in the REE estimation. Therefore, we propose that variabilities should be separately measured from their VO_2_ and VCO_2_ averages with the purpose to identify the monitors’ technological differences in the measurement of the variabilities and to search for possible physiological information in them during steady and non-steady IC studies. To prove this hypothesis, the VO_2_ and VCO_2_ averages and their variabilities were simultaneously measured by using the MC and the BbB techniques. Specifically, variances were used with the MC technique and power spectrum functions in the 0–0.5 Hertz band were constructed and measured in the BbB method. The gas exchange in the first method was sampled every 20 seconds while the gas exchange in the second technique was sampled breath by breath in order to generate a stochastic process measurement as a manner of a discrete time series. Both gas exchanges were measured in two consecutive 15 minute windows in order to obtain enough data to measure trends and to have high frequency resolution when exploring cyclical rhythms.

The objective of this work was to develop a pilot study where young healthy volunteer population was submitted to the physiological clino-orthostatic maneuver (COM) with the idea to generate steady/clinostatic and non-steady/orthostatic stages as a manner of an instrumental bench test (Gonzalez et al. 2013; Cadena et al. 2010). A hybrid calorimeter with the MC and the BbB techniques was applied to compare inter-individual variability changes during the COM stages. The inter-individual variabilities’ energy measurement was considered as appropriate since it can reflect physiological phenomena. Then, variance averages (*SD*_*VO*2_^2^, *SD*_*VCO*2_^2^) were used as time energy measurement in the MC technique and power spectrum functions (*vVO2*(*f*), *vVCO2*(*f*)) were used as frequency energy measurement in the BbB method. Finally, the inter-individual VO_2_ and VCO_2_ averages were computed with the purpose to compare the techniques’ performance in the REE estimation.

## Results and discussion

### Variabilities’ energy by power spectrum functions

**Figure 1:**
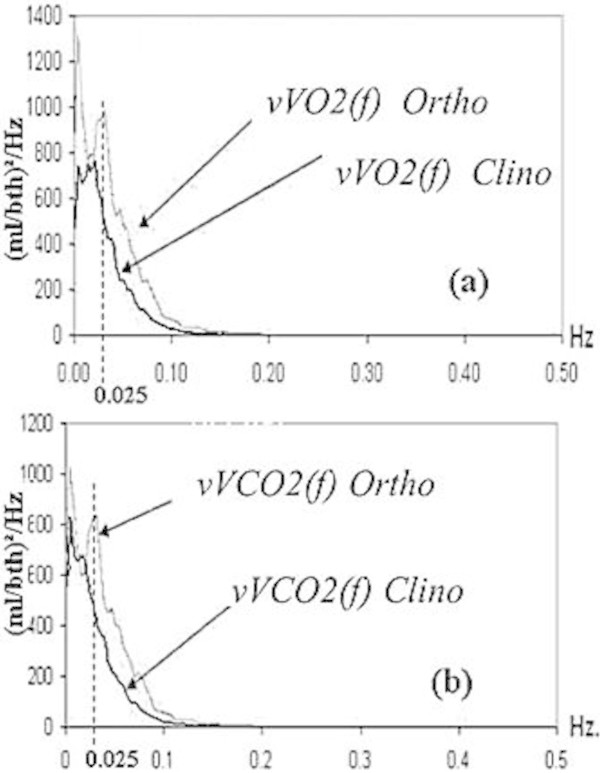
**The inter-subject variabilities analysis by power spectrums in the 0–0.5 Hertz frequency band.** The averaged power spectrum functions *vVO2*(*f*) in graph **(a)** and the function *vVCO2*(*f*) in graph **(b)** show a cyclic rhythm at 0.025 Hz only during the orthostatic stage of the COM.

The Figure 1 shows the BbB inter-subjects VO_2_ and VCO_2_ variabilities in terms of their averaged power spectrum functions *vVO2*(*f*) and *vVCO2*(*f*) with units in (ml/bth)^2^/Hz in the frequency range of 0–0.5 Hertz. Thus, individual spectrum functions were generated when each subject (N = 15) was submitted to the steady/clinostatic and then to the non-steady/orthostatic stages of the COM.

The averaged functions *vVO2*(*f*) and *vVCO2*(*f*) showed that their main energy was concentrated in the low frequency (LF) and medium frequency (MF) bands, where the analysis bands were defined as LF = 0–0.04, MF = 0.04–0.15 and HF = 0.15–0.50 Hertz. Here, it is noteworthy that the band definition for the variabilities was analogous to how the heart rate variability is analysed with the purpose to facilitate their physiological interpretation (Task Force of the European Society of Cardiology 1996). The Figure 1 clearly shows that the *vVO2*(*f*) and *vVCO2*(*f*) have an unexpected cyclic rhythm energy with central frequency at 0.025 Hz that is generated only during the non-steady/orthostatic stage of the COM with an energy increment of 71% and 56%, respectively, when this is compared with the steady state energy.

**Figure 2:**
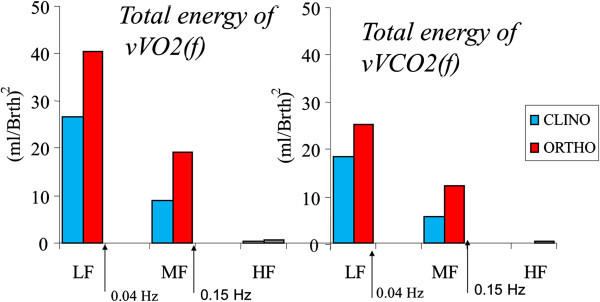
**The inter-subject total energy increments by frequency bands.** The increments in the total energy were 71% for the *vVO2*(*f*) and 56% for the *vVCO2*(*f*) due to the orthostatic stage.

The Figure 2 shows the variabilities’ total energy (ml/bth)^2^ increments by frequency bands. For instance, the total energy of *vVO2*(*f*) was computed as the integral below the function so that in orthostatic stage is approximately 50% higher in magnitude than the total energy of the *vVCO2*(*f*) for the same stage. A comparative analysis for the variability energies as a consequence of the COM application is shown in the Table 1.

**Table 1:** **Variability energy analyses by technique**

N = 15 subjects	(***SD***_***VO***2_)^2^MC (ml/bth)^2^	(***SD***_***VCO***2_)^2^MC (ml/bth)^2^	Total energy ***BbB*** (ml/bth)^2^	Total energy ***BbB*** (ml/bth)^2^
**STEADY/CLINO (SD)**	**3.81** (2.6)	**2.96** (2.1)	**35.6** (31.9)	**23.9** (24.5)
**UNSTEADY/ORTHO (SD)**	**3.92** (2.5)	**3.37** (4.0)	**60.2** (43.8)	**37.5** (35.2)
***t*** **-test**	**p** = 0.88	p = 0.77	**p < 0.05**	**p < 0.05**

Variances are for the MC while total energies are for the BbB techniques during the application of the clino-orthostatic maneuver.

Data in bold means averages and data in parenthesis means standard deviations.

### Comparative analysis of the variabilities’ energy

The Table 1 shows the comparative analysis of the variabilities’ energy obtained by the MC and the BbB techniques application. The variances averages (*SD*_*VO*2_)^2^ and (*SD*_*VCO*2_)^2^ are compared against the total variabilities’ averaged energy obtained from the *vVO2*(*f*) and *vVCO2*(*f*) functions.

The variance averages increment due to the COM were not statistically significant (p > 0.1), while the spectral total energy increment were 71% for *vVO2*(*f*) and 56% for the *vVCO2*(*f*) (p < 0.05) as it is also seen in Figure 2.

### Comparative analysis of averages

**Figure 3:**
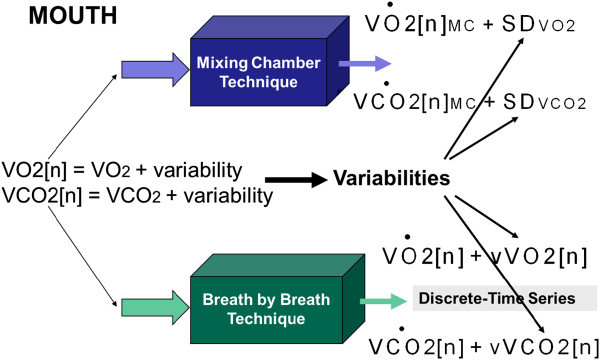
**The VO2 and VCO2 variabilities definition.** The variabilities are defined in terms of the MC and BbB techniques. The SDVO2 and SDVCO2 are for the mixing chamber (MC) and the vVO2[n], vVCO2[n] are for the breath by breath (BbB) techniques. The discrete gas exchange is defined at the mouth level as VO2[n] and VCO2[n] such that they include their own variabilities.

The Table 2 shows the VO_2_ and VCO_2_ average comparison according to the model of the Figure 3. A discrete gas exchange (*VO2*[*n*] and *VCO2*[*n*]) model was considered in order to compute separately the averages for the MC and BbB techniques. Then, the averages for the MC technique ( and *VĊO*2[*n*]_*MC*_) were incremented 63% and 39% (p < 0.05), while the averages for the BbB technique ( and *VĊO*2[*n*]) were only increased 32% and 40% (p < 0.1), when the COM was applied. The statistical differences analysis was based on the Welch *t*-test for unequal variances.

**Table 2:** **The MC and BbB averages analysis during the steady/clinostatic and non-steady/orthostatic stages**

N = 15 subjects	(ml/min)	***VĊO***2[ ***n*** ]_***MC***_(ml/min)	(ml/min)	***VĊO2[*** ***n*** ] (ml/min)
**STEADY/CLINO (SD)**	**167** (48)	**135** (42)	**152** (37)	**124** (36)
**UNSTEADY/ORTHO (SD)**	**273** (74)	**188** (70)	**202** (56.6)	**174** (64)
**Welch** ***t*** **-test**	**p < 0.05**	**p < 0.05**	**p < 0.1**	**p < 0.1**

Data in bold means averages and data in parenthesis means standard deviations.

**Figure 4:**
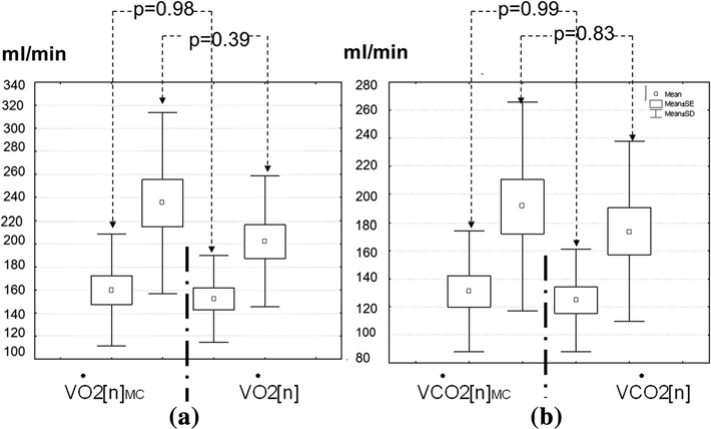
**Comparative analysis of averages between techniques.** The  and the *VĊO*2[*n*]_*MC*_ averages correspond to the MC technique whereas the  and  averages correspond to the BbB method. The graphs **(a)** and **(b)** do not show statistical differences between computed averages for both IC techniques.

Although all computed averages in Table 2 show independent significant statistical differences due to the COM, the Figure 4 shows no differences between the IC techniques. The post-hoc statistical analysis corroborates none differences (p > 0.1) using a Welch *t*-test for the inter-individual VO_2_ and VCO_2_ averages, after applying an ANOVA one-factor test for multiple measurements. However, it is worth to observe that the difference between the  and the  averages shows a slight tendency (p = 0.39) to reject the null hypothesis only during the orthostatic stage.

## Discussion

In view of the results, the hypothesis is proved in the sense that it is necessary to analyze the VO_2_ and VCO_2_ variabilities in order to understand the causes of variations in the REE estimation. The Table 1 shows the inter-individual variabilities’ energies computed as variances which did not show statistical differences during the COM test. These unchanged variances lead to the interpretation that the MC technique is a better method to perform IC studies with patients in steady and non-steady state since the REE is much less affected by the VO_2_ and VCO_2_ variabilities. The MC technique works as a low pass filter such that it suppresses the high frequencies of the variabilities generating more adequacies to measure the averages of the VO_2_ and VCO_2_. Thus, the analysis of averages in Table 2 is consequent with the MC technique performance when an increment of 63% for the  is observed while the  only shows an increment of 32%. Additionally, this result can be interpreted as the MC technique having the right sensitivity to faithfully follow any physiological low frequency change that affects the VO_2_ and VCO_2_ averages (Bruce 1996).

These outcomes lead us to understand why the old instrument Delta Track II (Datex Finland) has been accepted as the reference instrument when new metabolic monitors are compared against its performance, mainly during IC studies in critical care patients. It is clear that the canopy in the Delta Track II performs as an open circuit MC technique with the capability to reject high frequency variabilities. Deltra Track’s hardware can be modelled as a time average filter so that the VO_2_ and VCO_2_ variabilities from unstable patients produce minimum inter-individual variations in the REE estimation (Severine et al. 2013; Miodownik et al. 2000).

On the other hand, the power spectrum functions in Figure 1 show how IC studies in steady or non-steady state can be separated using their energy computation. The inter-individual variabilities’ energies in Table 2 show increments of 71% for the *vVO2*(*f*) and 53% for the *vVCO2*(*f*) when subjects are submitted to the orthostatic stage. These results suggest, the BbB technique is more suitable for monitoring and controlling the subjects’ physiological condition. This is an improvement over only using the traditional concept of CV. In addition, the cyclical rhythms found at 0.025 Hz in Figure 1 can be used as a new figure of merit to describe how stable or unstable the patient is during an IC study. Although this finding can be used as new physiological control information, the interpretation of its origins needs more research work. One first approach was carried out by dividing the energy of the *vVO2*(*f*) and *vVCO2*(*f*) in frequency bands similarly to the way that heart rate variability (HRV) is processed; after which, one second step would be to correlate the energy found in LF and MF with the LF energy of the HRV in order to discard whether or not the rhythmicity is due to sympathetic neural control or not (Taylor et al. 2001; Satue and Méndez 2012).

## Conclusions

New and advanced IC metabolic monitors designs should consider the following issues: (a) the VO_2_ and VCO_2_ variabilities should be separately measured using the MC and BbB techniques simultaneously in order to carry on IC studies in steady or non-steady state and to distinguish the origin of the variation of the REE estimation. (b) The MC technique is the appropriate method to estimate the VO_2_ and VCO_2_ averages, whereas the BbB technique is the most suitable procedure to provide physiological information to determine how stable or unstable an IC study is. (c) The cyclical rhythm in the BbB technique cannot be interpreted as instrumental noise since the O_2_ and CO_2_ transducers are performing independent measurements in open circuit calorimeters that allow to compute the *vVO2*(*f*) and *vVCO2*(*f*) separately. (d) The discrete gas exchange modeling of the Figure 3 contributes to understand why the MC technique is a low-pass filter or a moving average measurement system while the BbB technique is a high-pass filter able to generate a random process measurement system. Therefore, both techniques have different effects in the REE estimation. (e) Finally, it is important to notice that the COM physiological test combined with the VO_2_ and VCO_2_ variability measurements can be considered as an input–output paradigm assessment in order to search for the metabolic monitors’ performance.

## Methodology

### Model for the MC and BbB variabilities and averages definition

The Figure 3 shows a hybrid calorimeter model with the MC and the BbB techniques where the VO_2_ and VCO_2_ variabilities are defined at the output as *SD*_*VO2*_ and *SD*_*VCO2*_ and *vVO2*[*n*] and *vVCO2*[*n*], respectively. Likewise, the averages are defined as  and *VĊO*2[*n*]_*MC*_ for the MC technique and  and *VĊO*2[*n*] for the BbB method.

**Figure 5:**
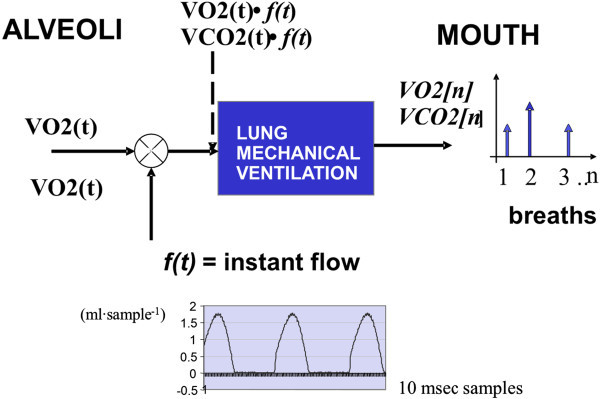
**The alveoli gas exchange model.** It is assumed that the VO2(t) and VCO2(t) are sampled by the lung’s mechanical ventilation to generate the discrete VO2[n] and VCO2[n] at the mouth level.

This model for the MC and BbB techniques considers a discrete gas exchange at the input of the mouth (*VO2*[*n*] and *VCO2*[*n*]) in which the variabilities are implicitly included before they are separately measured. The argument n = 1,2,3… stands for a discrete time series that represents the breath by breath gas exchange during an IC study. The model in Figure 5 explains how the discrete gas exchange is formed at the alveolar level.

### Model for the alveolar discrete gas exchange

The alveolar discrete gas exchange is modelled in Figure 5. The assumption is that the continuous gas exchange at the alveoli (*VO2*(*t*) and *VCO2*(*t*)) is sampled by the lung’s mechanical ventilation. Then a discrete gas exchange *VO2*[*n*] and *VCO2*[*n*] is generated when the breath by breath instant flow *f*(*t*) works as a sampling function as in Equations (1) and (2).12

Where: *f* (*t*) is the instant expired flow (L/sec). *V*_*T*_ is the tidal volume (ml) without BTPS (body, temperature, pressure, saturated) to STPD (standard, temperature, pressure, dry) volumetric corrections in order to preserve the simplicity of the model, *FIO*_2_(*t*) – *FEO*_2_(*t*) is the inspired-expired oxygen fraction difference and the *FECO*_2_(*t*) is the expired CO_2_ gas fraction. All gas fractions are in atmospheric percentages (%).

The products *f*(*t*) × *VO2*(*t*) and *f*(*t*) × *VCO2*(*t*) generate the continuous sampling for the O_2_ and CO_2_ uptake during each expired breath with time duration *D*_*1*_, *D*_*2*_, …*D*_*n*_. Hence, individual and different breath-by-breath sample volumes are produced as *VO2*[*Dn*] and *VCO2*[*Dn*]. These volumes are computed as in Equations (3) and (4). The instantaneous products are done between signals analog to digital converter (A/D) at the rate of 10 milliseconds per sample in order to avoid numerical integration errors and to be according with the sampling Nyquist theorem when it is assumed signals with bandwidths below 100 Hz (Proakis and Manolakis 1998).34

**Figure 6:**
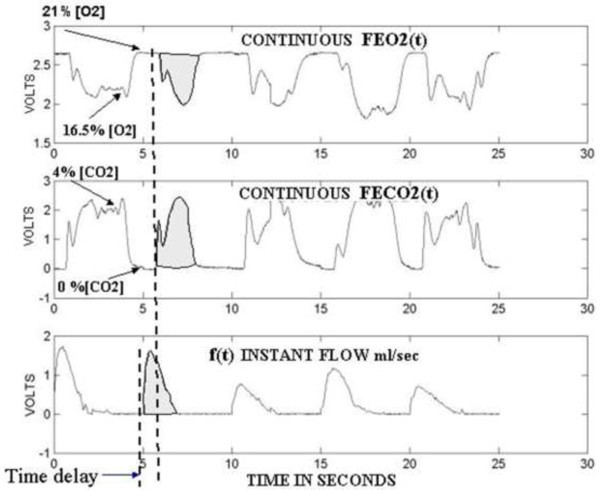
**Gas exchange fractions and instant flow signals.** The constant 800 millisecond time delay is considered in order to synchronize the computation for each *VO2*[*Dn*] and *VCO2*[*Dn*] and the corresponding time series generation.

**Figure 7:**
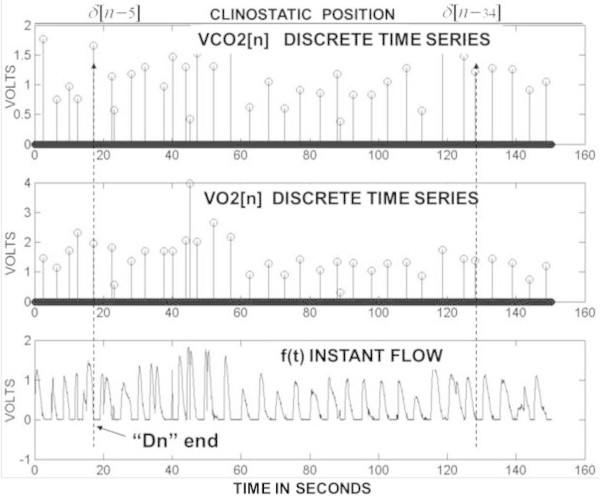
**The VO2[n] and VCO2[n] a discrete time series examples.** Two simultaneous values of VO2[Dn] and VCO2[Dn] are computed at δ[n-5] and δ[n-34]. The value of each Dn = 1,2, ..,N is placed at the end of the corresponding expired instant flow.

The *g* (*t* – *D*_*n*_) are continuous gate functions with the same time duration D_1_, D_2_,..D_n_ that allow synchronization to integrate the products between the instant flow and the instant gas fractions as it is seen in Figures 6 and 7. Normalized products *f*(*t*) × *VO2*(*t*) and *f*(*t*) × *VCO2*(*t*) are needed in order to match with the *f*(*t*) peak amplitude the *VO2*(*t*) and *VCO2*(*t*) values so that Equations (3) and (4) should be divided by *1*/*V*_*T*_. Figure 6 shows a real example how gas fractions signals and the expired instant flow signal are synchronized to compute each *VO2*[*Dn*] and *VCO2*[*Dn*].

### BbB discrete time series analysis

The *VO2*[*n*] *and VCO2*[*n*] discrete time series in Figure 7 are generated when the computations of each *VO2*[*Dn*] and *VCO2*[*Dn*] are carried out over the continuous signal outputs corresponding to the flow, O_2_ and CO_2_ sensors. The constant time delay of 800 msec in Figure 6 is for the synchronization between the instantaneous flow *f*(*t*) and the time gas fraction signals (*FEO2*(*t*) *and FECO2*(*t*)). This time lag depends on the sensors’ time response and the delay produced by the tubing length which utilizes a flow of 150 ml/min to sample the sensors. The hybrid calorimeter with the open pneumatic circuit is sketched in Figure 9. Each *VO2*[*Dn*] and *VCO2*[*Dn*] value is placed in a time series using the sequence *δ*[*n*] with mathematical proprieties that allow the generation of the BbB discrete time series according to Equations (5) and (6).56

Then, the sequence *δ* [*n* – *D*_*n*_] is used to geometrically place each value of *VO2*[*Dn*] and *VCO2*[*Dn*] as a series of coefficients at the end of each *f*(*t*) as it is seen in Figure 7. Here, the meaning of *Dn* is extended as a dumb variable (*Dn* = 1,2,..n) just to be interpreted as an index to generate the BbB discrete time series *VO2*[*n*] and *VCO2*[*n*]. The Figure 7 shows an example of a discrete time series from which the *νVO2*[*n*] and *νVCO2*[*n*] variabilities are computed. The average values ( and *VĊO*2[*n*]) are calculated from the discrete gas exchange as in Equations (7) and (8).78

Where:910

The above averages are computed with approximately N = 225 breaths, which are equivalent to a data acquisition window of 15 minutes.

**Figure 8:**
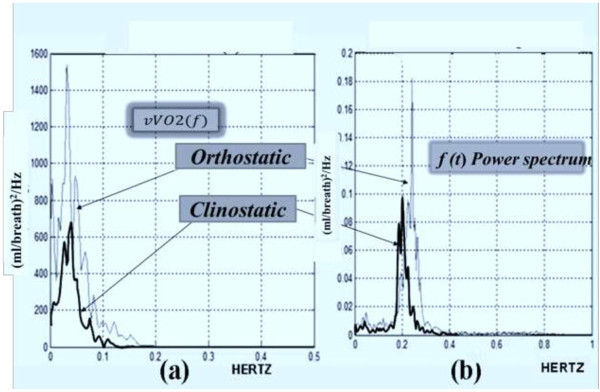
**Example of a** ***vVO2*** **(** ***f*** **) power spectrum in (ml/breath)** ^**2**^ **/Hz.** A subject underwent the clino-orthostatic maneuver. Graph **(a)** shows the *vVO2*(*f*) outlined in black that corresponds to the clino stage. The power spectrum function outlined in gray corresponds to the orthostatic stage. Graph **(b)** shows the power spectra for the continuous instant flow *f*(*t*).

An example of the power spectrum analysis of the *νVO2*[*n*] and *νVCO2*[*n*] is shown in the Figure 8. A linear data interpolation function was used to reformat the discrete time series *VO2*[*n*] and *VCO2*[*n*]. Then, one sample per second was used to resample the reformatted discrete time series in order to obtain a frequency domain analysis in the range of 0.0 to 0.5 Hz. The processing window was selected to capture at least 15 minutes of data so that a Welch power spectrum estimator allowed a maximum resolution of 0.005 Hertz using 50% of data overlapping. The frequency band analysis was defined in three main regions: low frequencies (LF = 0–0.04 Hz), medium frequencies (MF = 0.05–0.15 Hz) and high frequencies (HF = 0.16–0.5 Hz). These band divisions are similar to the heart rate variability analysis with special emphasis in the LF and MF bands since the HF band is assumed to be related with instant flow’s frequency (respiratory frequency) activity as it is seen in the example of the Figure 8.

### Averages and variabilities in the MC technique

The measurement of the averages and variabilities using the MC technique requires modeling the effect of the mixing chamber upon the discrete gas exchange *VO2*[*n*] and *VCO2*[*n*], having the model in Figure 3 in mind. The MC averages should be computed as in Equations (11) and (12) using a digital moving average which depends on the chamber volume and the number of breaths that the chamber storages as a manner of pipe-line, prior to obtaining one sample average every 20 seconds. In our case, the hybrid calorimeter has a chamber with a volume of 1.8 Liters so that the number of breaths storage in the MC, when the patient’s respiratory frequency is approximately 15 breaths/min, is approximately M = 4 in Equations (11) and (12). And the  and *VĊO*2[*n*]_*MC*_ are computed using the criteria of 30 averages to smooth enough the gas exchange. Thus, each one of the 30 averages is formed with M breaths to obtain one average sample during a total of 15 minutes per each IC study.1112

The measurement of the variabilities in the MC technique was computed as in Equations (13) and (14). Even, these equations allow the calculation of the VCs according to the clinical practice guidelines as it is shown in (15).131415

### Hybrid indirect calorimeter hardware

**Figure 9:**
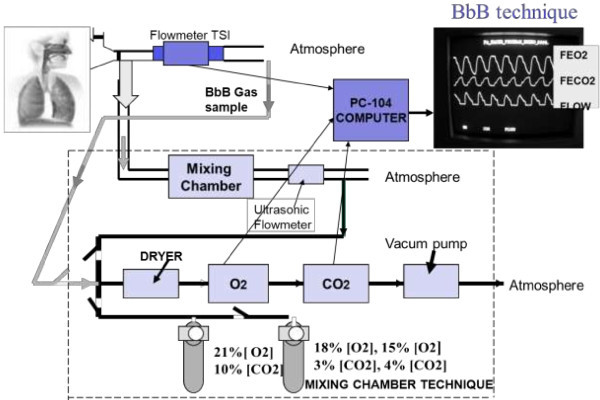
**A block diagram of the hybrid calorimeter MGM-3 is shown.** The MC section is outlined in dotted lines and the blue blocks point out the BbB pneumatic open circuit section. The PC-104 is a dedicated computer to obtain real time data from the flow meter, the O_2_ and CO_2_ sensors.

A specific open-circuit hybrid indirect calorimeter (MGM-3) was designed and manufactured for the purpose of this work which was based in the design of Westenskow et al. (1984). The MC and the BbB techniques were fused in the MGM-3 as it can be seen in Figure 9. The patient’s half mask works either by passing the expired gas through the 1.8 L mixing chamber to implement the MC technique or by directly connecting the expired gas to a hot-wire flowmeter (TSI Inc, USA) to implement the BbB technique.

The MGM-3 calibration and quality control unit was a microprocessor based design and was calibrated every 5 minutes using a reference gas cylinder with a certified mixture of 21% O_2_, 10% CO_2_, complemented with N_2_. Additionally, two more gas certified mixture cylinders (15% O_2_, 4% CO_2_ and 18% O_2_, 3% CO_2_, Praxair) were used to adjust the transducer offsets and gains for the case of doing IC studies in ambulatory patients. The MC technique was implemented by displaying values of V_E_ (expired volume minute in L · min^-1^), RF (respiratory frequency in breaths · min^-1^), VO_2_ (ml · min^-1^), VCO_2_ (ml · min^-1^)_,_ V_T_ (tidal volume in ml · breath^-1^) and RQ (respiratory quotient VO_2_/VCO_2_) every 20 seconds. These readings were automatically corrected and displayed at STPD conditions after measuring volumes and fractions at BTPS conditions (2400 meters above the sea level at Mexico City, 590 ± 3 mmHg, and expired gases’ temperature). The VO_2_ was computed using the Haldane correction.

### Experimental design and data processing

A population of 15 young normal volunteer subjects without a history of any chronic disease was studied. The ages ranged from 18 to 30 years with a body mass index (BMI) average of 24.2 ± 3.8 Kg · m^-2^. All subjects gave signed informed consent to be studied in the morning after 8 hours of fasting. Subjects were asked to perform the active clino-orthostatic maneuver (COM). First, a 5 minute period of relaxing was used before he/she lied down on a couch and was then submitted to the COM while connected to the MGM-3 calorimeter. Two 30 minutes periods were used to implement the measurement protocol: 15 minutes for the MC technique and 15 min for the BbB technique in each COM position. All of the measurements were made in the same room maintaining constant temperature and data collection by the same expert team in all cases.

Comparative statistical paired data analysis was applied intra-groups. The MC averages  and *VĊO*2[*n*]_*MC*_ were compared against the BbB averages  and . Similarly, variances averages (*SD*_*VO*2_)^2^ and (*SD*_*VCO*2_)^2^ were compared against total spectral energy averages. The statistics analysis was parametric since the variables were considered to be Gaussians, once they were tested by the Kolmogorov–Smirnov test. Then, two-tailed paired t-tests (Welch version) were used as appropriate for unequal variances. In all cases, the null hypothesis was rejected when p ≤ 0.1 since this experiment was considered to be a pilot study.

## Abbreviations

IC: Indirect calorimetry
COM: Clino-orthostic maneuver
REE: Resting energy expenditure (Kcal/day)
VO_2_: Oxygen consumption (ml/min)
VCO_2_: Dioxide production (ml/min)
MC: Mixing chamber technique
BbB: Breath-by-breath technique
VC: Variation coefficient (SD/average)
SD: Standard deviation
SD^2^VO2: VO_2_ variance (energy) (ml/breath)^2^
SD^2^VCO2: VCO_2_ variance (energy) (ml/breath)^2^
vVO2(f): VO_2_ variability average power spectrum function (ml/breath)^2^/Hz
vVCO2(f): VCO_2_ variability average power spectrum function (ml/breath)^2^/Hz
Total energy: (ml/breath)^2^
VO_2_ average by the MC technique (ml/min)
VĊO2[n]MC: VCO_2_ average by the MC technique (ml/min)
VO_2_ average by the BbB technique (ml/min)
VĊO2[n]: VCO_2_ average by the BbB technique (ml/min)
VO2[n] and VCO2[n]: Discrete gas exchange at the mouth
VO2(t) and VCO2(t): Continuous gas exchange at the alveoli
vVO2[n]: VO_2_ time discrete series variability (ml/min)
vVCO2[n]: VCO_2_ time discrete series variability (ml/min).
